# Predictive value of spinal CSF volume in the preoperative assessment of patients with idiopathic normal-pressure hydrocephalus

**DOI:** 10.3389/fneur.2023.1234396

**Published:** 2023-10-05

**Authors:** Nenad Kudelić, Ivan Koprek, Milan Radoš, Darko Orešković, Ivana Jurjević, Marijan Klarica

**Affiliations:** ^1^Department of Neurosurgery, General Hospital Varaždin, Varaždin, Croatia; ^2^Department of Pharmacology, Croatian Institute for Brain Research, School of Medicine, University of Zagreb, Zagreb, Croatia; ^3^Department of Molecular Biology, Ruđer Bošković Institute, Zagreb, Croatia; ^4^Department of Neurology, University Hospital Centre Zagreb, Zagreb, Croatia

**Keywords:** idiopathic normal-pressure hydrocephalus, CSF volume, cranial CSF, spinal CSF, prognostic test, preoperative assessment

## Abstract

**Introduction:**

The pathophysiology, diagnosis, and management of idiopathic normal pressure hydrocephalus (iNPH) remain unclear. Although some prognostic tests recommended in iNPH guidelines should have high sensitivity and high predictive value, there is often no positive clinical response to surgical treatment.

**Materials and methods:**

In our study, 19 patients with clinical and neuroradiological signs of iNPH were selected for preoperative evaluation and possible further surgical treatment according to the guidelines. MR volumetry of the intracranial and spinal space was performed. Patients were exposed to prolonged external lumbar drainage in excess of 10 ml per hour during 3 days. Clinical response to lumbar drainage was assessed by a walk test and a mini-mental test.

**Results:**

Twelve of 19 patients showed a positive clinical response and underwent a shunting procedure. Volumetric values of intracranial space content in responders and non-responders showed no statistically significant difference. Total CSF volume (sum of cranial and spinal CSF volumes) was higher than previously published. No correlation was found between spinal canal length, CSF pressure, and CSF spinal volume. The results show that there is a significantly higher CSF volume in the spinal space in the responder group (*n* = 12) (120.5 ± 14.9 ml) compared with the non-responder group (103.1 ± 27.4 ml; *n* = 7).

**Discussion:**

This study demonstrates for the first time that CSF volume in the spinal space may have predictive value in the preoperative assessment of iNPH patients. The results suggest that patients with increased spinal CSF volume have decreased compliance. Additional prospective randomized clinical trials are needed to confirm our results.

## 1. Introduction

Normal-pressure hydrocephalus (NPH) is a form of chronic communicating hydrocephalus characterized by enlarged ventricles with normal or occasionally slightly elevated intracranial pressure. NPH can be of unknown cause (idiopathic; iNPH) or secondary if known causes such as intracranial hemorrhage, trauma, central nervous system infections or intracranial neurosurgical procedures lead to its development (1, 2).

iNPH is a chronic steadily progressing disease. It is the most common form of hydrocephalus in older adults and usually occurs in people over 60 years of age, equally in men and women (2, 3). Its differential diagnosis to other neurodegenerative diseases is often difficult due to variable clinical manifestations. The number of patients with iNPH has been steadily increasing, most likely due to improved health care and increased longevity (4). As the volume of cerebrospinal fluid (CSF) inside the brain gradually increases at the expense of the brain parenchyma, characteristic neurological symptoms (so-called Hakim's or Adams triad-urinary incontinence, balance disorders and dementia) develop (5, 6). iNPH should be considered as a diagnosis for patients with unexplained symmetric gait disturbance, a frontal-subcortical pattern of cognitive impairment, and urinary urge incontinence, whose MRI scans show enlarged ventricles and whose comorbidities are not sufficient to explain their symptoms.

In general, pathophysiological mechanism of hydrocephalus development is often not clear in both experimental animals and patients. This especially applies to particular forms of the disease such as unilateral communicating hydrocephalus and hydrocephalus conjoined with spinal pathology (7, 8), as well as transitory hyrocephalus [e.g., after intracranial bleeding; (9–12)], arrested or slow- progressing forms with normal CSF pressure and uninterrupted CSF pathways such as iNPH (8, 13, 14). Since these conditions cannot be explained with classical concept of CSF physiology and pathophysiology (blocked CSF system communication, imbalance between CSF secretion and absorption), etiology and pathogenesis of the mentioned forms of the disease still remain poorly understood.

Despite its unclear nature iNPH is considered by many as a treatable cause of dementia and ataxia because a significant number of patients may improve clinically with ventricular shunting (15–19). Conventional definition of iNPH implies “symptomatic improvement achieved by CSF shunt placement”. However, since this outcome can only be measured postoperatively, the definition is unsuitable for the preoperative diagnosis of iNPH (13). Permanent CSF diversion via shunt insertion represents a gold standard in iNPH treatment. Response to the shunting for properly selected patients is about 85% (18). Thus, these results arise a question: is it possible to predict which patients will respond to treatment?

The first step consists of being certain about correct diagnosis. However, despite current guidelines, the diagnostic criteria for iNPH are still unclear. Various invasive prognostic tests, such as tap test (large-volume lumbar puncture), prolonged external lumbar drainage with gait testing before and after CSF removal or CSF infusion testing for measurement of CSF outflow resistance are used to identify patients who are likely to respond to shunting surgery (13). It has not been established whether the continuous CSF drainage test is more predictive of the efficacy of a shunt intervention than the CSF tap test. If both the typical symptoms (gait disturbance, cognitive impairment and urinary incontinence) and radiological findings are present, a shunt intervention is likely to improve the symptoms in the absence of other comorbidities (13, 14, 17, 20).

Although some of the prognostic tests recommended in the iNPH guidelines should have high sensitivity and high predictive value, sometimes there is no positive clinical response to surgical treatment. Therefore, there are probably other factors that can significantly influence clinical outcome, one of them being the state of regional cerebral blood flow (CBF) before and after surgery. Recent studies in which non-invasive radiological techniques have been applied (i.e., arterial spin labeling MRI, resting-state functional MRI) clearly demostrated importance of regional CBF determination in preoperative iNPH patient selection (21, 22).

Preoperative evaluation of iNPH patients usually involves lumbar drainage, which removes a certain volume of CSF from the CSF system without determining the overall starting CSF volume (both cranial and spinal). Indeed, from the existing literature data, it can be concluded that the values of total CSF volume vary considerably (14). Thus, it appears that during external CSF drainage, a decrease in CSF volume resulting in decreased CSF pressure, as well as a change brain perfusion pressure, would be different in each individual patient. It is believed that the positive effect of the tap test is due to the removal of metabolites that affect cerebral perfusion. For these reasons, in addition to the usual diagnostic procedures, in our study we also measured cranial and spinal CSF volumes before lumbar drainage and recorded the changes in CSF pressure, as well as in clinical status, caused by the CSF extraction. Because there is no general consensus on the exact volume of CSF that should be removed from the system (it's usually removed until the lumbar pressure drops to zero), it would be interesting to determine whether removal of the same volume causes similar changes in all patients.

## 2. Materials and methods

This study was conducted at the Department of Neurosurgery, General Hospital Varaždin, Croatia in the period from January 2017 to August 2019.

### 2.1. Ethics statement

The conducted study was voluntary and all data used during the study were previously protected, anonymized and stored in a database. The study protocol was approved by Ethics Committee of the Faculty of Medicine, University of Zagreb: October 30, 2017 (No: 380-59-10106-20-111/78, Class 641-01/20-02/01), and Ethics Committee of the General Hospital Varaždin: January 20, 2017 (Number: 02/1-91/79-2017). The patients provided their written informed consent to participate in this study.

### 2.2. Subjects

Subjects were patients with clinical and neuroradiological signs of iNPH that came to our clinic for possible shunting procedure evaluation. Patient selection for neurosurgical treatment (ventriculoperitoneostomy) was performed according to the latest iNPH guidelines (13, 23). In 19 patients, lumbar puncture, measurement of lumbar CSF pressure in the lateral recumbent position and MR imaging of the neural axis were performed. Clinical outcome was measured by the Mini-Mental State Examination (MMSE) test and the 10-m walk test (10 MWT). Subjects with previous surgical interventions within the craniospinal space associated with a proven intracranial or spinal tumor and/or inflammatory process were excluded from the study (potential secondary NPH). After a positive clinical response to prolonged external lumbar drainage, 12 patients underwent VP shunting procedure.

### 2.3. MRI

Patients underwent MRI on GE 1.5T Signa HDXT (GE, USA) or Siemens Magnetom Aera 1.5 T (Siemens, Germany) device. Brain MRI was performed in standard sequences and additional high-resolution sagittal T1 sequences of the head were taken for research purposes. The first MR device 1.5T GE Signa HDXT using FSPGR 3D (fast spoiled gradient echo) recorded T1 sequences (TR = 9.9 ms; TE = 3.9 ms; resolution 256 × 256; voxel size 1 × 1 × 1 mm) and the second MR device 1.5 T Siemens Magnetom Aera recorded T1-MPRAGE (magnetization prepared rapid gradient echo) sequences (TR = 2.2 ms; TE = 2.6 ms; resolution 256 × 240; voxel size 1 × 1 × 1 mm).

Additionally, the first MR device recorded T2 3D Myelo sequences (TR = 3,000 ms; TE = 598 ms; resolution 288 × 192, voxel size 1.4 × 1.4 × 1.8 mm) of the spine, while the second MR device recorded 3D-T2 SPACE sequences (TR = 3,000 ms; TE = 435 ms; resolution 320 × 320; voxel size 1.25 × 1.25 × 1.3 mm) of the spine.

High spatial resolution sequences were used, which provide a good contrast between the CSF and the surrounding tissue, i.e., enable clear detection of the edges of the cerebrospinal fluid space. MR imaging of the spine was performed in two segments due to its length: the upper segment includes the craniocervical junction, cervical and thoracic spine up to the T8-T9 level, while the lower segment includes thoracic spine from the T7 level to the sacrum. With this method of recording, the entire neural axis was covered and the resulting overlapping regions in the craniocervical and thoracolumbar areas were excluded from the volume calculation by computer processing of the images.

### 2.4. Volumetric analysis of MR images

**Figure 1:**
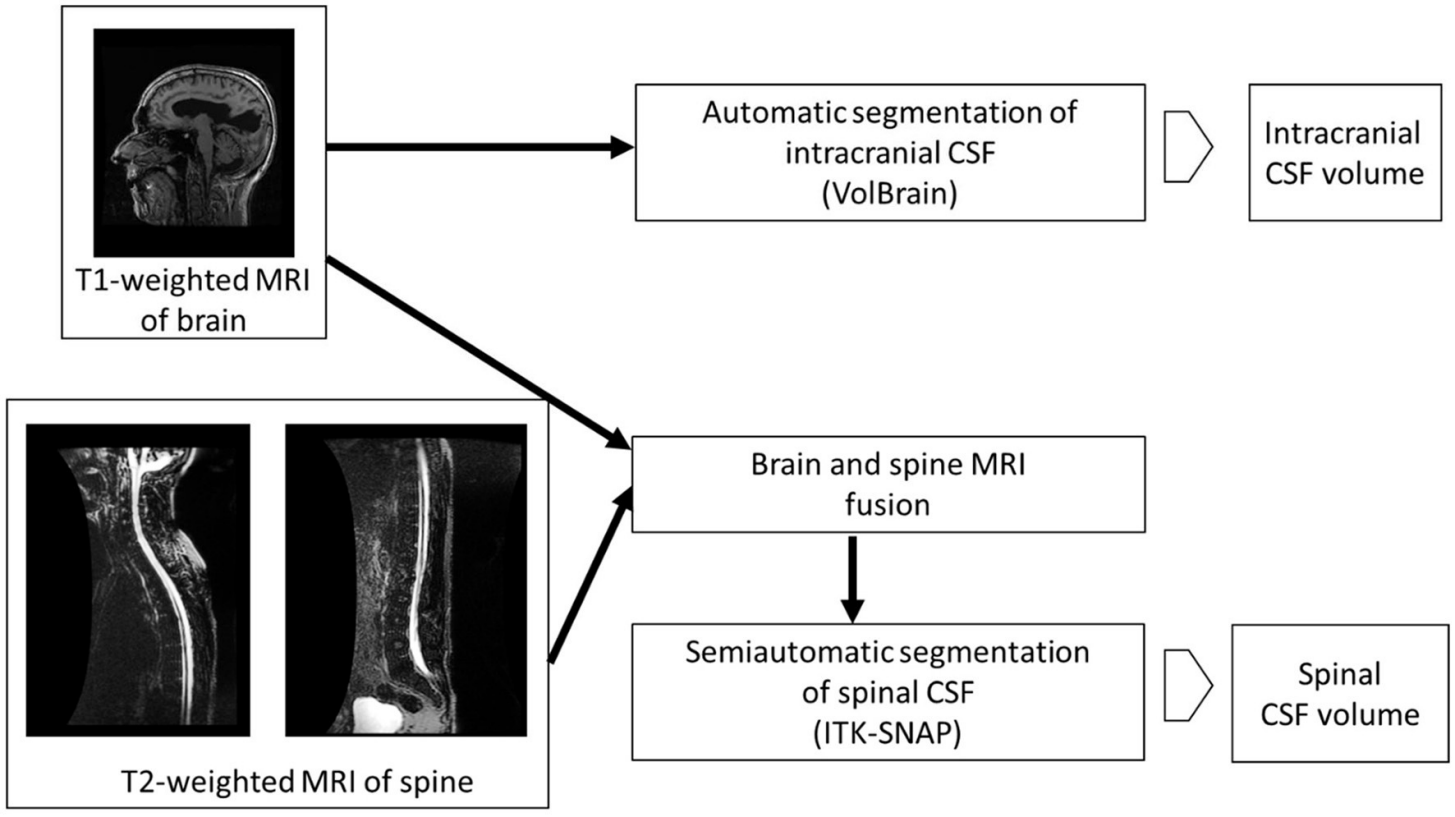
Volumetric analysis of brain and spine MR images.

Volumetric analysis of intracranial CSF was performed using the online platform volBrain (24). After anonymization of the images of the head DICOM MR with the MicroDicom Viewer program (http://www.microdicom.com), conversion to NIfTI format was performed with the dcm2nii program tool, which is part of the free MRICRON program package (https://www.nitrc.org/projects/mricron). The images were then compressed in zip format and uploaded to the volBrain platform. The volBrain system generates a data report in PDF format containing information about the volume of the intracranial cavity (ICC) and its parts (CSF, gray matter, white matter), and information about some macroscopic areas such as cerebral hemispheres, cerebellum, and brainstem by processing anonymized data in NIfTI format. After automatic segmentation of the subcortical structures, their volumes are determined (Figure 1).

**Figure 2:**
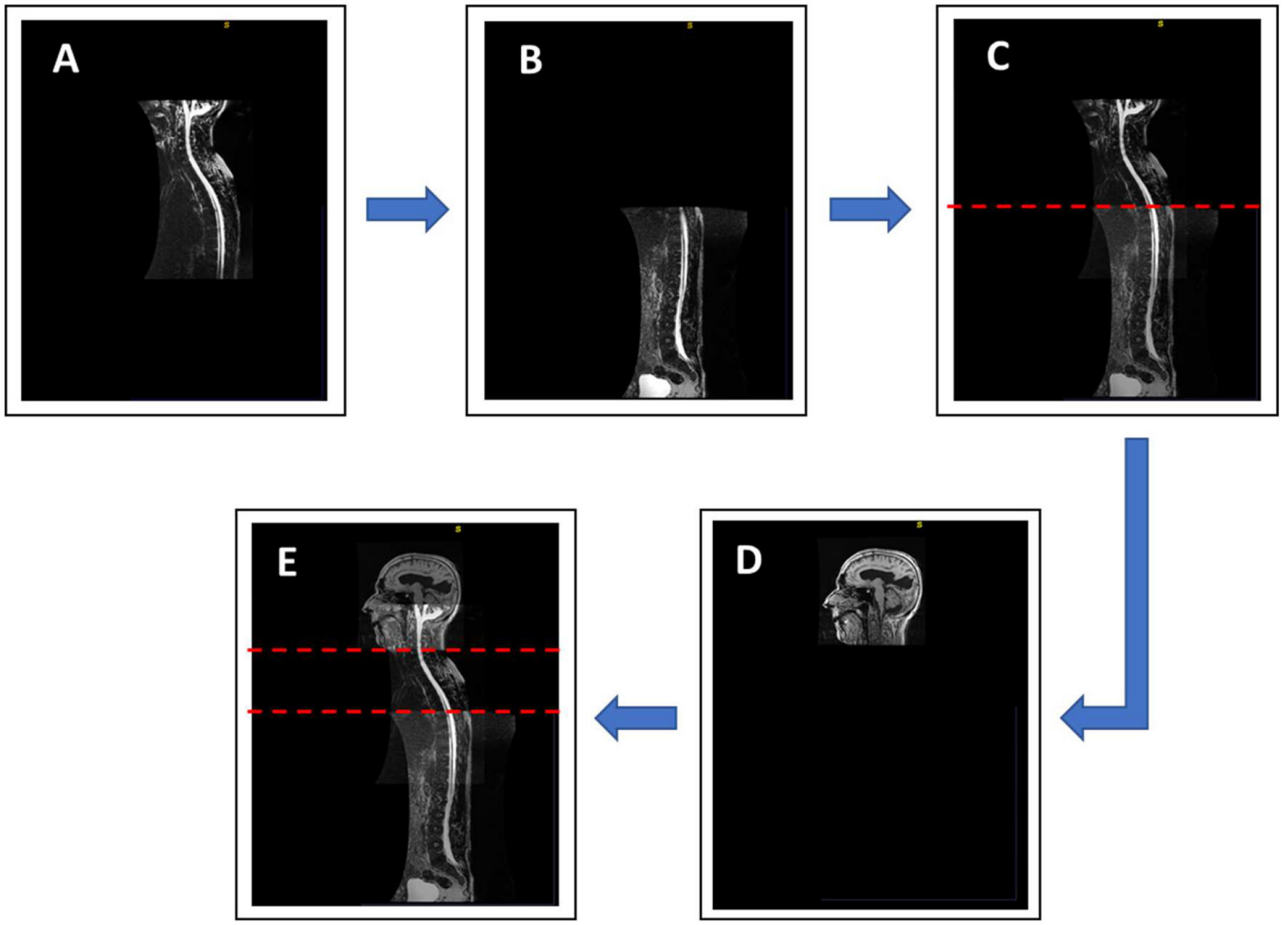
Sagittal image **(E)** showing complete coverage of the neural axis, obtained by MRI superposition and fusion of three different regions: cranial T1 images **(D)**, cervicothoracic **(A)** and thoracolumbar **(B)** T2 images of spinal segments. **(C)** Image of the spine obtained by fusion of two recorded segments of the spine. The red dashed lines show overlapping of individual segments.

Although methods for automatic segmentation of the spinal space have been described in the literature (25), reliable methods for automatic segmentation of CSF in the spinal compartment do not yet exist. Therefore, semi-automatic segmentation was used in this study. Prior to the actual segmentation, a superposition and fusion of T1 images of the head and T2 images of the cervicothoracic and thoracolumbar regions of the spine were performed (Figure 2). The lowest point of automatic segmentation of the intracranial space was determined, and segmentation of the spinal space was performed from this level. Overlapping regions were considered in the volumetry (Figure 2).

Segmentation of the spinal space was performed using the program ITK-SNAP v3.8.0 (http://www.itksnap.org). Before data processing, the patient's DICOM images were anonymized using the MicroDicom Viewer program, and then converted to the NIfTI file format. After automatic contrast adjustment, the region of interest (ROI) is defined, and in the pre-segmentation procedure the edges of the spinal canal and spinal cord are detected using the threshold mode.

Spinal segmentation was done separately for the cervicothoracic and thoracolumbar segment, taking into account the overlapping regions in the craniocervical and thoracolumbar part of the neural axis obtained by fusion of images of the cranium and the spine. The volumes of the cervicothoracic and thoracolumbar segments are shown collectively as a single volume of the spinal CSF.

The length of the spinal space was measured on MR scans from the level of the craniocervical junction (foramen magnum) to the end of the dural sac in the area of the sacral spinal canal.

### 2.5. Lumbar puncture, drainage and CSF pressure measurement

In patients with clinical and neuroradiological signs of iNPH, preoperative testing by CSF drainage was performed. In practice, usually two methods of testing are performed: lumbar puncture with large volume discharge (TAP test or large volume lumbar puncture—LVLP) or prolonged CSF drainage via lumbar drain. In this study, CSF pressure in lateral recumbent position before and after extraction of 15 ml of CSF was measured.

The lumbar drain was inserted, lumbar drainage was opened and continuous release of the CSF was set at a rate of 10 ml/h (about 250 ml/day) for the next 3 days. During the entire test period, the patient's clinical condition was monitored by measuring cognitive and walking scores (MMSE, 10 MWT).

### 2.6. Statistics

Descriptive statistical analysis was used to analyze the values of lumbar CSF pressure and CSF volumes obtained by automatic and semi-automatic segmentation of the head and spine MR images. Values of the measured variables are presented in contingency tables, while the values of continuous variables are presented in the corresponding parameters—arithmetic mean and standard deviation. Comparation of the arithmetic means was examined by the *t*-test for related samples. The parametric Pearson and non-parametric Spearman methods were used to examine the relationship between the two variables. Differences confirmed at the level of *p* < 0.05 were considered statistically significant.

## 3. Results

In 19 patients with iNPH, MRI of the brain and entire spine was performed, followed by volumetric analysis of the cranial and spinal space. After a positive clinical response to prolonged external lumbar drainage (improvement in Mini-Mental Test score and prolonged walking distance), 12 patients underwent surgery (responders). There was no statistical difference in age between the responder (seven male and five female; age 70.7 ± 6.4 years) and non-responder group (three male, four female; age 72.3 ± 6.3 years).

### 3.1. Cranial MRI volumetry

Table 1 shows results of cranial MRI volumetry of proportion of white matter, gray matter and cerebrospinal fluid, volumes of intracranial and ventricular CSF, total intracranial volume and the cerebral volume in all patients. In two patients, the volumetric system (volBrain) could not calculate cranial volumes due to a large deviation of anatomical characteristics from normal (large ventricles). Both patients were in non-responder group. The total volume of CSF in the cranial space in both groups (responders and non-responders) was 305.1 ± 103.9 ml (mean ± SD; *n* = 17). A comparison of the volumetric values of the intracranial space contents in responders and non-responders shows that there is no statistically significant difference between the two groups (*p* < 0.05).

**Table 1:** The table shows aggregated data of intracranial content of iNPH patients (total; responders; non-responders) obtained by the on-line volumetric system volBrain.

	**volBrain**		
	**Total (*****n*** = **17)**	**Responders (*****n*** = **12)**	**Non-responders (*****n*** = **5)**
WM %	41.4 ± 13.5	42.7 ± 13.8	38.3 ± 13.6
GM %	38.1 ± 9.1	37.3 ± 8.5	40.0 ± 11.1
CSF %	20.5 ± 6.1	20.0 ± 6.4	21.8 ± 5.6
CSF ventr (ml)	102.3 ± 45.0	102.3 ± 47.0	102.3 ± 44.9
CSF cranial (ml)	305.1 ± 103.9	297.3 ± 108.5	323.8 ± 101.0
IC (ml)	1,474.8 ± 188.6	1,476.5 ± 213.7	1,470.7 ± 129.3
BRAIN (ml)	1,169.7 ± 163.5	1,179.2 ± 190.2	1,146.9 ± 81.2

Data are presented as mean ± standard deviation (WM %, percentage of white matter; GM %, percentage of gray matter; CSF %, percentage of intracranial cerebrospinal fluid; CSF ventr, volume of ventricular cerebrospinal fluid in ml; CSF cranial, volume intracranial cerebrospinal fluid in ml; IC, intracranial volume in ml; BRAIN, volume of the cerebrum in ml).

### 3.2. Spinal MRI volumetry

**Figure 3:**
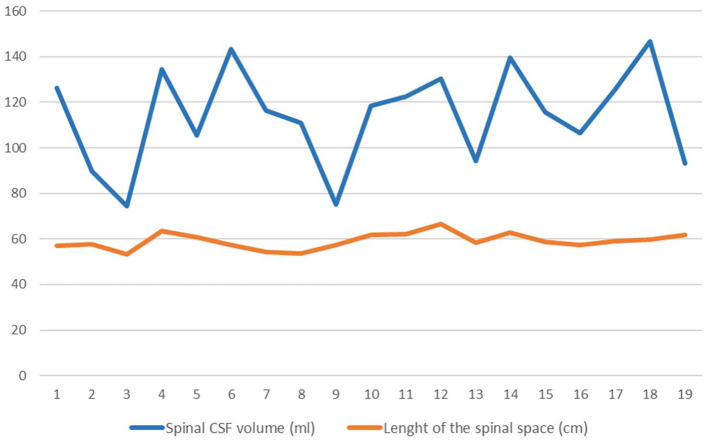
Values of length of the spinal canal (orange line) in cm and the spinal CSF volume (blue line) in ml. Lines connect points representing each patient's individual value (*n* = 19).

Anatomical parameters of the spinal space (length of the spinal space and volume of spinal CSF) were analyzed from MR images (Figure 3).

The volume of the spinal CSF in all patients was 114.1 ± 21.4 ml on average, and the length of the spinal space was 59.1 ± 3.5 cm. Statistical analysis of the data indicates that in our sample there is no correlation between the volume of CSF in the spinal space and the length of the spinal space.

**Figure 4:**
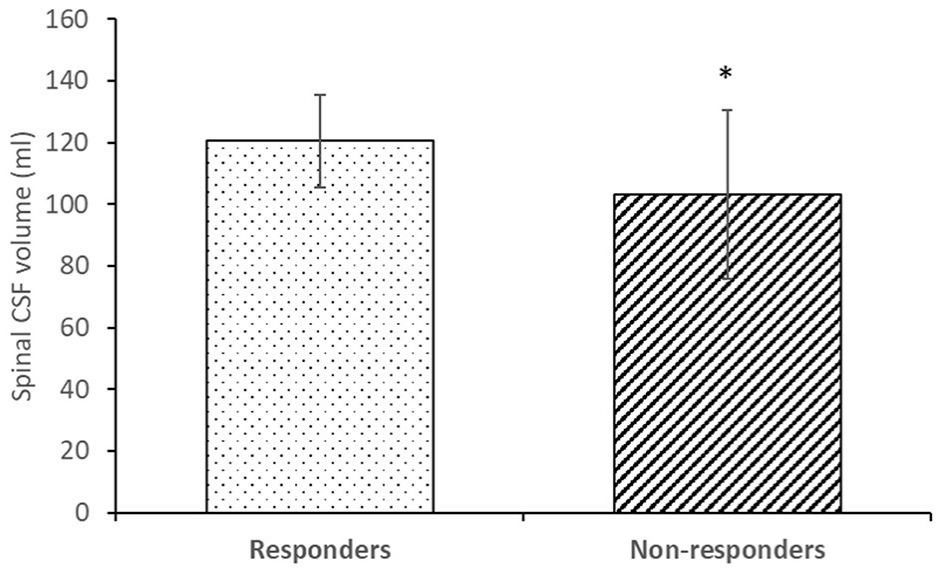
Spinal CSF volume in responders (*n* = 12) and non-responders (*n* = 7). Columns represent the mean value, and vertical lines SD. * *p* < 0.05.

Figure 4 shows a comparison of the CSF volume in the spinal space in the group of responders and non-responders. The volume of spinal CSF in the responder group (120.5 ± 14.9 ml) was statistically significantly different (*p* < 0.05) than in the non-responder group (103.1 ± 27.4 ml).

### 3.3. CSF pressure/CSF volume

The correlation between the lumbar CSF pressure measured in the lateral recumbent position and the CSF volumes was examined. The data show (Table 2) that CSF pressure, and total CSF volume values varied from 7.3 to 21.2 cm H_2_O, and from 254.8 to 594.1 ml, respectively.

**Table 2:** iNPH patient's individual value of lumbar CSF pressure (cm H_2_O) measured in the lateral recumbent position, lumbar CSF pressure (cm H_2_O) after 15 ml of CSF extraction, volume of cranial CSF (ml), volume of spinal CSF (ml), total volume of CSF (ml) obtained by MR volumetry and clinical response (n, negative and r, positive clinical response to prolonged external lumbar drainage).

**No**.	**CSF pressure (cm H_2_O)**	**CSF pressure (cm H_2_O); −15 ml**	**Cranial CSF volume (ml)**	**Spinal CSF volume (ml)**	**Total CSF volume (ml)**	**Clinical response**
1	15.0	8.0	398.8	126.3	525.1	n
2	20.0	11.0	297.5	89.9	387.4	n
3	20.0	8.0	^*^	74.4	^*^	n
4	18.7	10.0	361.2	134.5	495.6	r
5	17.3	12.0	350.3	105.5	455.8	r
6	20.7	11.2	111.4	143.4	254.8	r
7	13.0	14.7	190.9	116.4	307.3	n
8	20.5	11.0	165.1	110.8	275.9	r
9	18.0	13.6	284.4	75.1	359.5	n
10	15.0	9.7	137.8	118.2	256.0	r
11	21.1	14.6	259.3	122.5	381.8	r
12	18.8	14.2	428.7	130.4	559.1	r
13	16.2	11.8	298.5	94.2	392.7	r
14	7.3	6.0	317.5	139.6	457.1	r
15	14.5	15.7	339.0	115.5	454.5	r
16	11.5	8.5	357.6	106.4	464.0	r
17	18.7	16.9	441.1	125.6	566.6	r
18	18.1	14.7	447.4	146.7	594.1	n
19	21.2	16.4	^*^	93.2	^*^	n
mean ± SD	17.1 ± 3.7	12.0 ± 3.1	305.1 ± 103.9	114.1 ± 21.4	422.8 ± 108.4	

^*^In two patients, the volumetric system could not calculate cranial CSF volume.

A comparison of the intracranial, spinal or total CSF volumes with the level of lumbar CSF pressure measured in lateral recumbent position shows that there is no statistically significant correlation between lumbar CSF pressures and CSF volumes (*p* < 0.05).

**Figure 5:**
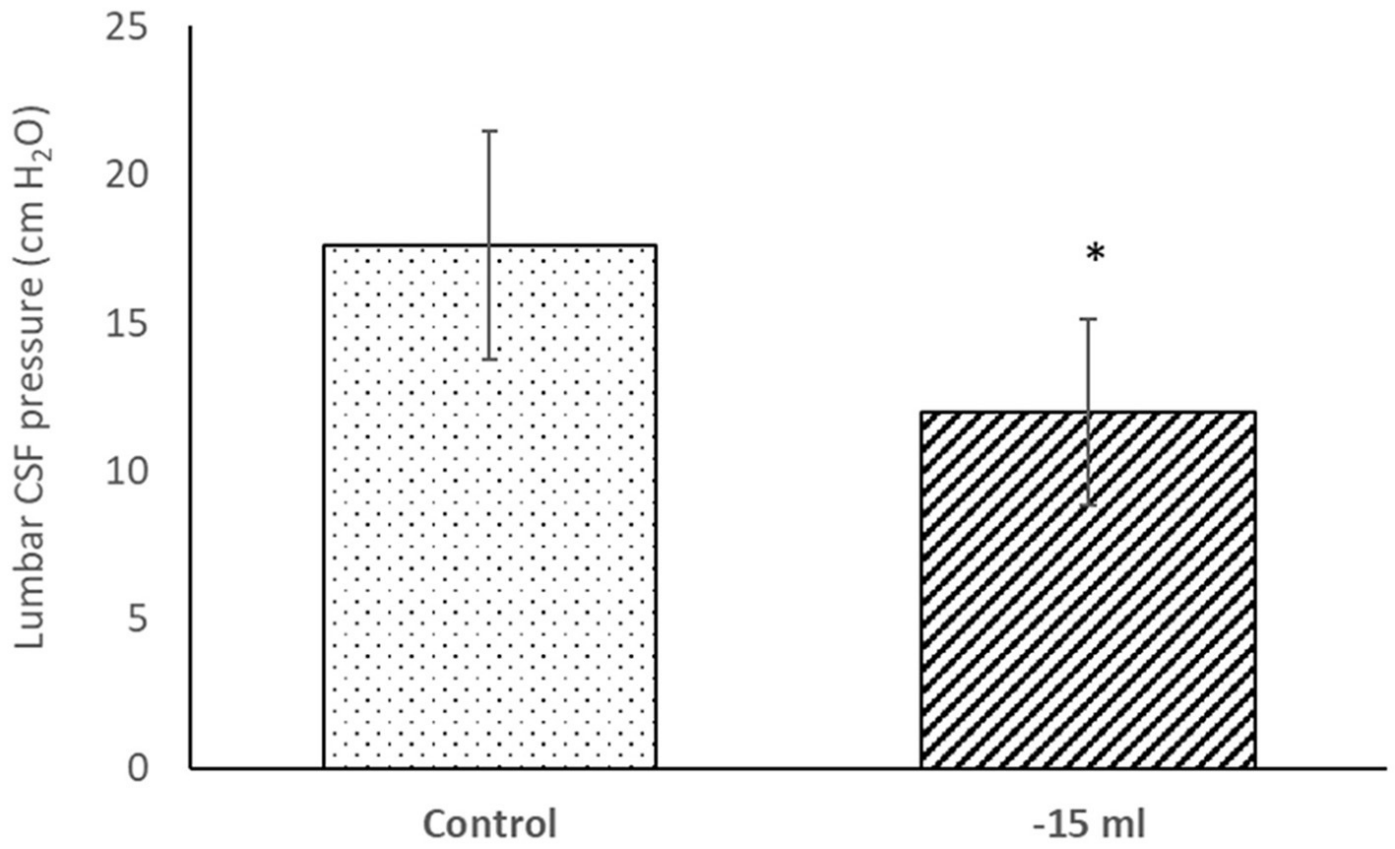
Lumbar CSF pressure (cm H_2_O) in lateral recumbent position before (Control) and after (−15 ml) removal of 15 ml of CSF by lumbar puncture. Results are presented as mean (column) ± SD (vertical line) (*n* = 19). *p < 0.05.

After removal of 15 ml of CSF from lumbar subarachnoid space by lumbar puncture, there was a statistically significant drop (*p* < 0.05) of the lumbar CSF pressure from 17.1 ± 3.7 cm H_2_O to 12.0 ± 3.1 cm H_2_O (mean ± SD; *n* = 19) (Figure 5). There is no statistically significant difference in the drop of lumbar CSF pressure after removal of 15 ml CSF between the responder (11.8 ± 3.1 cm H_2_O) and non-responder group (12.3 ± 3.4 cm H_2_O).

## 4. Discussion

In this study of 19 patients with iNPH, we performed MR volumetry of the entire craniospinal space during the diagnostic preoperative procedure. The results of this study show that the group of patients who responded positively clinically to prolonged external lumbar drainage did not differ from the patients who showed no improvement in terms of standard radiological parameters measured (volumes of brain tissue, intracranial space, cranial CSF, and ventricular CSF) within the cranial space (Table 1) and CSF pressures measured in the lateral recumbent position (Table 2). However, there was a significant difference in CSF volumes measured in the spinal subarachnoid space (Figure 4). The radiologic analysis of the cranial space in our study is consistent with a systematic literature review and meta-analysis (26) performed to identify radiologic features that can be used to distinguish iNPH shunt responders from non-responders. In the 27 articles included in the analysis, there was no single imaging parameter that differed between responders and non-responders. However, our study suggests that spinal CSF volume could be a new important prognostic factor for patients with iNPH.

### 4.1. Variability of CSF volume in cranial and spinal space

It was previously believed that the total CSF volumes in infants vary from 40 and 60 ml, in older children between 60 and 100 ml, while it is approximately 150 ml in adults (27–29). The total volume of the brain ventricles in adults was believed to be about 25–30 ml. Many recent studies suggest that the total CSF volume is much higher (14), i.e., ~150 ml or more in the intracranial subarachnoid space, between 100 and 120 ml in the spinal subarachnoid space, and additional 100–300 ml in the interstitial fluid of the central nervous system interstitial fluid (30–34).

Analysis of the CSF volume data obtained in our study shows that the total CSF volume is much higher than previously published. CSF volume in the cranium varied between 111.4 and 447.4 ml (mean 305.1 ± 103.9 ml) (Tables 1, 2), whereas the mean ventricular volume was 102.3 ± 45 ml (Table 1). Thus, in our iNPH patient group, the ventricular volume of ventricles was ~70 ml larger than the normal volume (25–30 ml). If we add the spinal CSF volume, which varied from 74.4 to 146.7 (mean, value 114.1 ± 21.4 ml; Table 2), to the cranial CSF volume, we can see that the total CSF volume varied from 254.8 to 594.1 ml (mean, 422.8 ± 108.4 ml; Table 2). If we subtract 70 ml from the mean total CSF volume because of hydrocephalus, the value of total CSF volume in potentially healthy individuals is still much higher than previously thought. Since there was no apparent correlation between spinal CSF volume and length of the spinal canal (Figure 3), the question arises as to what is the normal variability of spinal volume variability, and with that, what is the normal variability of total CSF volume.

### 4.2. Cranial and spinal compliance in iNPH patients

Compliance (C) is the ratio between the change in volume (ΔV) and the change in pressure (ΔP) and is usually described by an equation: C = ΔV/ΔP. Thus, in a highly compliant system, a large increase in volume results in only a small change in pressure, while in a less compliant system, a small increase in volume can result in a large increase in pressure. The compliant brain can accommodate a substantial increase in ventricular volume with a small increase in intracranial pressure. In clinical studies, compliance of the entire craniospinal space is usually determined, because compliance of the cranial and spinal spaces could only be determined separately if the cranial space were experimentally separated from the spinal space (35). In our study, no correlation was found between CSF pressure values in iNPH patients in the horizontal position, which varied within the normal range (from 7.3 to 21.2 cm H_2_O; Table 2), and values of total, cranial, or spinal CSF volume, which varied between 254.8 amd 594.1 ml, 111.4 and 447.4 ml, and 74.4 and 146.7 ml, respectively (Table 2). From this, we can logically conclude that it is not clear what factors determine CSF volume, CSF pressure, compliance, and other parameters that are important for patient monitoring and that have been thus far extensively researched in the physiology and pathophysiology of the CSF system.

Withdrawal of 15 ml CSF during lumbar puncture resulted in a decrease in CSF pressure in our patients (Table 2; Figure 5), although this CSF pressure drops varied widely, ranging from almost insignificant changes to a maximum of 12 cm H2O. These acute pressure changes did not correlate with either initial CSF volumes or clinical outcomes, so the pressure value does not appear to have predictive value.

Many studies reveal that spinal space is crucial as a compensatory space for the regulation of CSF pressure and subsequently, brain perfusion. We have recently shown experimentally that the addition or extraction of a volume of fluid significantly alters intracranial CSF pressure in both horizontal and upright positions without significantly altering cranial CSF volume (35). A similar observation was also made in the study of patients in whom automated MRI measurements of craniospinal CSF volume were performed. It was found that after extraction of CSF from the lumbar space, there was a decrease in intracranial pressure, which was mainly related to the increase in spinal compliance rather than cranial compliance, as reduced spinal CSF volume was observed while cranial CSF volume remained almost unchanged (36, 37). These clinical observations, in which significant changes in spinal CSF volume occurred without significant changes in cranial CSF volume, support the new concept of intracranial fluid behavior and CSF pressure regulation, according to which a relatively rigid intracranial space prevents sudden changes in cranial neurofluid volumes, thus maintaining brain perfusion during changes in body position and low blood pressure (35, 38). The results of our previous studies (35, 38–40) suggest that the role of the spinal subarachnoid space in regulating CSF pressure is pivotal and is consistent with research findings that focus on the spinal rather than the intracranial space in compensating for intracranial hypertension (37, 41, 42).

According to all of the afore mentioned, it is likely that in our study, after lumbar puncture and an acute decrease in CSF volume of 15 ml, there was primarily a spinal decrease in CSF volume (without significant change in cranial CSF volume), associated with a decrease in CSF pressure throughout the CSF system, and an improvement in cerebral perfusion. Thus, we would expect that LP shunts reduce the spinal CSF volume, while VP shunts keep the cranial and spinal CSF volume in the physiological range. This would be in accordance with the clinical findings in patients with spinal CSF leak, who do not tolerate the upright position well, and in those with cranial CSF leak, who do not tolerate the horizontal position well. The detailed explanation of this effect was published in our previous papers (35, 38).

This would also be consistent with studies using MRI to examine cerebral perfusion before and after the tap-test (43, 44). In the recent years, global and regional CBF has been assessed (perfusion and diffusion MRI performed) in patients with iNPH and it was observed that CBF was reduced in iNPH patients compared to healthy controls with a clear correlation between the clinical improvement and increase of CBF (after tap test) (45–48). One of the most recent studies in which non-invasive tehnique of arterial spin-labeling (ASL) perfusion MRI (21) was used and ASL colored maps analyzed before and after tap test demonstrated good correlation of CBF and clinical improvement which suggested that this tehnique could aid and support the invasive tests in preoperative selection for shunt surgery of possible iNPH patients. Additional recent study on 32 iNPH patients in which resting-state functional MRI (22) was used to explore blood oxygenation level-dependent signal fluctuations at rest in different areas of the brain before and after infusion and tap test suggested the presence of partially reversible plasticity functional mechanism in interhemispheric, frontal, occipital, default-mode and motor network circuits, which provides new possibilities for adequate presurgical patient selection.

Increased spinal CSF volume would selectively mean that the main compensatory compartment is almost exhausted. Our results suggest that patients with an average greater CSF volume responded well clinically to lumbar drainage. It appears that lumbar drainage in their case increased compliance, i.e., the compensatory capacity of the entire craniospinal space, and regulated CSF pressure fluctuations, thereby improving brain perfusion. Previously, it was observed that the decrease in ICP wave amplitude (indirect indicator of compensatory capacity) during external lumbar drainage was a powerful indicator (49). In patients with decreased spinal CSF volume, the compensatory capacity does not appear to be nearly exhausted regardless of the potential causes of hydrocephalus development, which may explain why drainage procedure did not significantly alter the interrelationship between volumes and pressure of neurofluids (blood, interstitial fluid, CSF) inside their craniospinal space.

### 4.3. Classic and novel understanding of neurofluid physiology and pathophysiology

According to the classical concept, there is constant CSF secretion, unidirectional circulation, and predominantly passive absorption through arachnoid granulations into the dural brain sinuses (31, 50, 51). The development of hydrocephalus is also mainly explained as a consequence of an imbalance between CSF secretion and absorption. Until today, no reliable method has been developed to accurately measure CSF formation or absorption, as all existing methods (vetriculo-cisternal perfusion-indirect method, direct perfusion method; phase-contrast MRI; time-spatial labeling inversion pulse; arterial spinlabeling MRI) have large discrepancies in measurements (52–54). The classical view is that most of the CSF within the head is reabsorbed by the arachnoid granulations along the dural venous sinuses (85–90%). The smaller portion is thought to be absorbed along the spinal nerves root sheaths (10–15%) (14, 34). Some tests have been used to draw indirect conclusions about the quality of CSF reapsorption (e.g., infusion test). Some authors demonstrated CSF absorption abnormality in iNPH using such an infusion test (55), so it is assumed that impaired CSF reabsorption could be due to abnormalities at the level of the arachnoid villi or the interstitial extracellular space, or both (55, 56). CSF absorption, which is predominantly via arachnoid granulation, has recently been challenged by an MRI study showing that a person with a normal CSF system before 10 years of age has no arachnoid granulation, whereas in adults aged 20–80 years, a larger number of individuals have no or few granulations (57).

The results of our study in iNPH patients suggest that the infusion test would probably indicate abnormal absorption in patients with large spinal CSF volume, as the infusion would cause a significant increase in pressure due to decreased compensatory capacity.

The classical concept that iNPH is simply a form of CSF circulation disorder, in which there is an imbalance between CSF production and reabsorption, is probably not valid (7, 8, 58). Indeed, it has been found that there is a net retrograde movement of CSF from the subarachnoid space through the aqueduct into the ventricles in a large number of patients without hydrocephalus, as well as in almost 80% of patients with communicating hydrocephalus (53, 59, 60). Using phase-contrast MRI, severe fluid fluctuations and large net flow (outside normal physiologic values) through the aqueduct were additionally observed, along with a significant extracranial (spinal) contribution of CSF to the CSF water content (59).

The pathophysiology of iNPH is associated with impaired distribution of metabolites in the interstitium of the brain and, in general, with impaired blood circulation. Thus, it appears that in iNPH, impaired glymphatic circulation may lead to brain tissue damage and eventually dementia (61). It appears that the cerebral macrovascular and microvascular network is critical for clearance of interstitial fluid and removal of other dissolved substances such as amyloid- beta, while their damage may worsen cerebral perfusion, neurofluid movement, and drainage of waste products, leading to neuronal and glial degeneration (40, 62). This is consistent with the new understanding of neurofluid (blood, interstitial, and cerebrospinal fluid), physiology which views interstitial and cerebrospinal fluid as a functional unit whose volume is regulated by the gradient between osmotic and hydrostatic forces at the level of the brain and spinal cord capillary network (54, 63).

Periventricular white matter perfusion decreases with age due to various pathological processes such as atherosclerosis, hypertensive arteriolar sclerosis, and diabetes, which may decrease the efficiency of small-caliber perforating arteries and lead to deep white matter ischemia. Clinically apparent NPH could also be triggered by lacunar periventricular white matter infarctions due to abnormal CSF pulse dynamics (64, 65). Numerous factors could reduce shunt response, such as more severe preoperative symptoms, older age, and cerebrovascular disease related to diabetes mellitus, hypertension, hyperlipidemia, and history of myocardial infarction (66). In some iNPH patients who responded poorly to shunt therapy, a radiological association with primary neurodegenerative diseases such as AD and subcortical dementia can be established (62, 67). Conversely, it appears that good shunt- responders have a lower burden of neuritic plaques or intraventricular amyloid and tau (67).

## 5. Conclusion

Craniospinal space analysis based on iNPH patient MR images strongly suggests significantly larger values of CSF volume both inside the cranium and inside the spinal compartment from those available in the literature. No connection between the length of the spinal canal, the CSF pressure value and the measured spinal CSF volume has been detected. Larger mean spinal CSF volume is associated with positive clinical response to external lumbar drainage. It seems that patients with enlarged spinal CSF volume have decreased compliance as spinal space represents the key compensatory capacity of the entire craniospinal space.

## 6. Limitations

There are several limitations of this study. Our sample size was adequate for detecting the possible connection between average spinal CSF volume and a positive clinical response to extended external spinal CSF drainage. However, it should be noted that our predictive analysis asked for studies involving serial assessments in larger samples. Our study also did not include information regarding genetic factors (i.e., APOE genotype) nor comorbid medical conditions or medications patients were taking, which may have influenced the results. The larger sample size and additional data regarding their comorbidities would also enable us to investigate the possible correlation between spinal CSF volumes of patients with predominantly cognitive deficit and those with predominantly motor disturbance. In our future studies we aim to extend our investigation in that direction.

## Data availability statement

The original contributions presented in the study are included in the article/supplementary material, further inquiries can be directed to the corresponding author.

## Ethics statement

The studies involving humans were approved by Ethics Committee of the Faculty of Medicine, University of Zagreb, Croatia Ethics Committee of the General Hospital Varaždin, Croatia. The studies were conducted in accordance with the local legislation and institutional requirements. The participants provided their written informed consent to participate in this study.

## Author contributions

NK and MK designed and conceptualized the study. NK and IK conducted CSF pressure measurements and VP shunt operations. NK and MR performed MRI volumetry and calculated CSF volumes. MK, DO, NK, and IJ drafted the initial version of the manuscript and explained the presented results. All authors have read and approved the final draft of the manuscript.
